# Chalcogen passivation: an *in-situ* method to manipulate the morphology and electrical property of GaAs nanowires

**DOI:** 10.1038/s41598-018-25209-x

**Published:** 2018-05-02

**Authors:** Zai-xing Yang, Yanxue Yin, Jiamin Sun, Luozhen Bian, Ning Han, Ziyao Zhou, Lei Shu, Fengyun Wang, Yunfa Chen, Aimin Song, Johnny C. Ho

**Affiliations:** 1 Shenzhen Research Institute of Shandong University, Shenzhen, 518057 P. R. China; 2 Center of Nanoelectronics and School of Microelectronics, Shandong University, Jinan, 250100 P. R. China; 3 State Key Laboratory of Multiphase Complex Systems, Institute of Process Engineering, Chinese Academy of Sciences, Beijing, 100190 P. R. China; 4 Department of Materials Science and Engineering, City University of Hong Kong, 83 Tat Chee Avenue, Kowloon, Hong Kong; 5 Shenzhen Research Institute, City University of Hong Kong, Shenzhen, 518057 P. R. China; 6 College of Physics and Cultivation Base for State Key Laboratory, Qingdao University, Qingdao, 266071 P. R. China; 7 School of Electrical and Electronic Engineering, University of Manchester, Manchester, M13 9PL UK; 8 State Key Laboratory of Millimeter Waves, City University of Hong Kong, 83 Tat Chee Avenue, Kowloon, Hong Kong

## Abstract

Recently, owing to the large surface-area-to-volume ratio of nanowires (NWs), manipulation of their surface states becomes technologically important and being investigated for various applications. Here, an *in-situ* surfactant-assisted chemical vapor deposition is developed with various chalcogens (e.g. S, Se and Te) as the passivators to enhance the NW growth and to manipulate the controllable p-n conductivity switching of fabricated NW devices. Due to the optimal size effect and electronegativity matching, Se is observed to provide the best NW surface passivation in diminishing the space charge depletion effect induced by the oxide shell and yielding the less p-type (i.e. inversion) or even insulating conductivity, as compared with S delivering the intense p-type conductivity for thin NWs with the diameter of ~30 nm. Te does not only provide the surface passivation, but also dopes the NW surface into n-type conductivity by donating electrons. All of the results can be extended to other kinds of NWs with similar surface effects, resulting in careful device design considerations with appropriate surface passivation for achieving the optimal NW device performances.

## Introduction

Due to their superior carrier mobilities and tunable bandgaps, III-V compound semiconductors are widely investigated as the active channel materials for transistors beyond silicon complementary metal-oxide-semiconductor (CMOS) technology and highly efficient photovoltaics^1–10^. However, as the device scaling is aggressive for the transistor performance enhancement as well as for the improved high efficiency/cost ratio of solar devices (e.g. using non-crystalline glass substrates to reduce cost)^11^, the corresponding elevated surface-area-to-volume ratio of these miniaturized III-V materials (e.g. nanowires, NWs) would contribute large amounts of surface traps and/or states, which significantly modify their intrinsic electronic transport properties^12–19^. One such notorious phenomenon is known as Fermi level pinning, in which the substantial density of surface states give rise to a fixed barrier height (i.e. surface band bending) independent of the overlaying material. As a result, the surface Fermi level would be existed at a fixed location, contributing parasitic resistances to consume unnecessary energy during any device operation. In the meanwhile, these significant surface states would also deteriorate the carrier mobility by scattering and decrease the photo-to-electrical conversion efficiency by serving as recombination centers for the photo-generated electron/holes, degrading performances radically in transistors and solar cells, respectively^17,20–22^. Therefore, it is a great challenge and urgent issue to tackle the surface states for the low-dimensional III-V nanomaterials, such as the one-dimensional (1D) NWs, in order to alleviate the adverse surface effects on their electrical properties.

Until now, there are various technologies developed to manage the above-mentioned surface issues on NWs, including the surface passivation by Al_2_O_3_^23,24^ and sulphur^25–28^ etc., as well as the core/shell heterostructure formation by adding an inert shell material^29–34^. In specific, the surface states of GaAs NWs can be effectively passivated by immersing them into (NH_4_)_2_S solution to form a surface covalent sulfur bonding layer^28^ or overgrowing a crystalline GaN shell layer^33^. This way, the recombination rate of electron/holes pairs would be decreased and the resulting photovoltaic efficiency of fabricated GaAs NWs based devices would be enhanced accordingly^16,35,36^. Apart from GaAs NWs, the surface states will as well form an electron accumulation layer on the InAs NW surface, leading to surface Fermi level pinned above the conduction band and making the p-type conductivity challenging. Recently, crystalline InP shells have been successfully grown onto the InAs NW core to lessen the effect of Fermi level pinning in order to achieve the p-type conductivity there^37^. Nevertheless, all these passivation techniques still suffer from the need of *ex-situ* operating procedures (e.g. immersing fabricated NWs into solution) making the subsequent process integration questionable, or the sophisticated *in-situ* schemes (e.g. overgrowing NWs with crystalline shell layers) restricting practical utilization of NWs, it is highly desirable to develop facile, effective and versatile *in-situ* surface passivation methods for the further applications of III-V NWs.

In the past study, an *in-situ* sulfur surfactant assisted approach has been established in the chemical vapor deposition (CVD) of GaSb NWs, in which the S atoms can effectively passivate the reactive surface Sb constituents, facilitating the growth of thin (e.g. below 20 nm in the diameter), long and uniform GaSb NWs with the hole mobility approaching the theoretical limit^38–40^. On the other hand, the acceptor-like surface states, existed between intrinsic GaAs NWs and their surface native oxide shells, has also been utilized to control the electronic transport properties of GaAs NWs with different diameters. When the NW diameter decreases to <40 nm, the space-charge depletion layer, induced by the surface traps, would extend deeply into the NW core to deplete all electrons, leading to inversion and p-type conductivity as compared with the thick (>70 nm) intrinsically n-type GaAs NWs^19^. In this study, we adopt both the *in-situ* surfactant assisted CVD scheme employing different chalcogens, such as S, Se and Te, with different work functions as the passivator along with the manipulation of acceptor-like surface states, induced by the surface native oxide shells, to tailor the electronic transport properties of GaAs NWs. Interestingly, when chalcogens are employed as the *in-situ* passivator during the NW synthesis, their diameters become much thinner and more uniform, indicating the slowdown of the uncontrolled radial NW growth due to the proper surface passivation of reactive As constituents. Moreover, because of the optimal size effect and electronegativity, Se is found to provide a better surface passivation to the GaAs NWs and their parallel NW arrays in diminishing the space charge depletion effect induced by the native oxide shell, and yielding less p-type (i.e. inversion) or even insulating conductivity, as compared with S delivering intense p-type conductivity for the thin NWs with the diameter of ~30 nm. Te does not only provide the surface passivation, but also dopes the NW surface into n-type conductivity by donating electrons owing to its lower electronegativity than the one of As. All these designate the effectiveness of controllable p-n switching of thin GaAs NWs by *in-situ* chalcogen surface passivation, which is promising to further III-V nanomaterials for next-generation electronics and optoelectronics.

## Results

**Figure 1:**
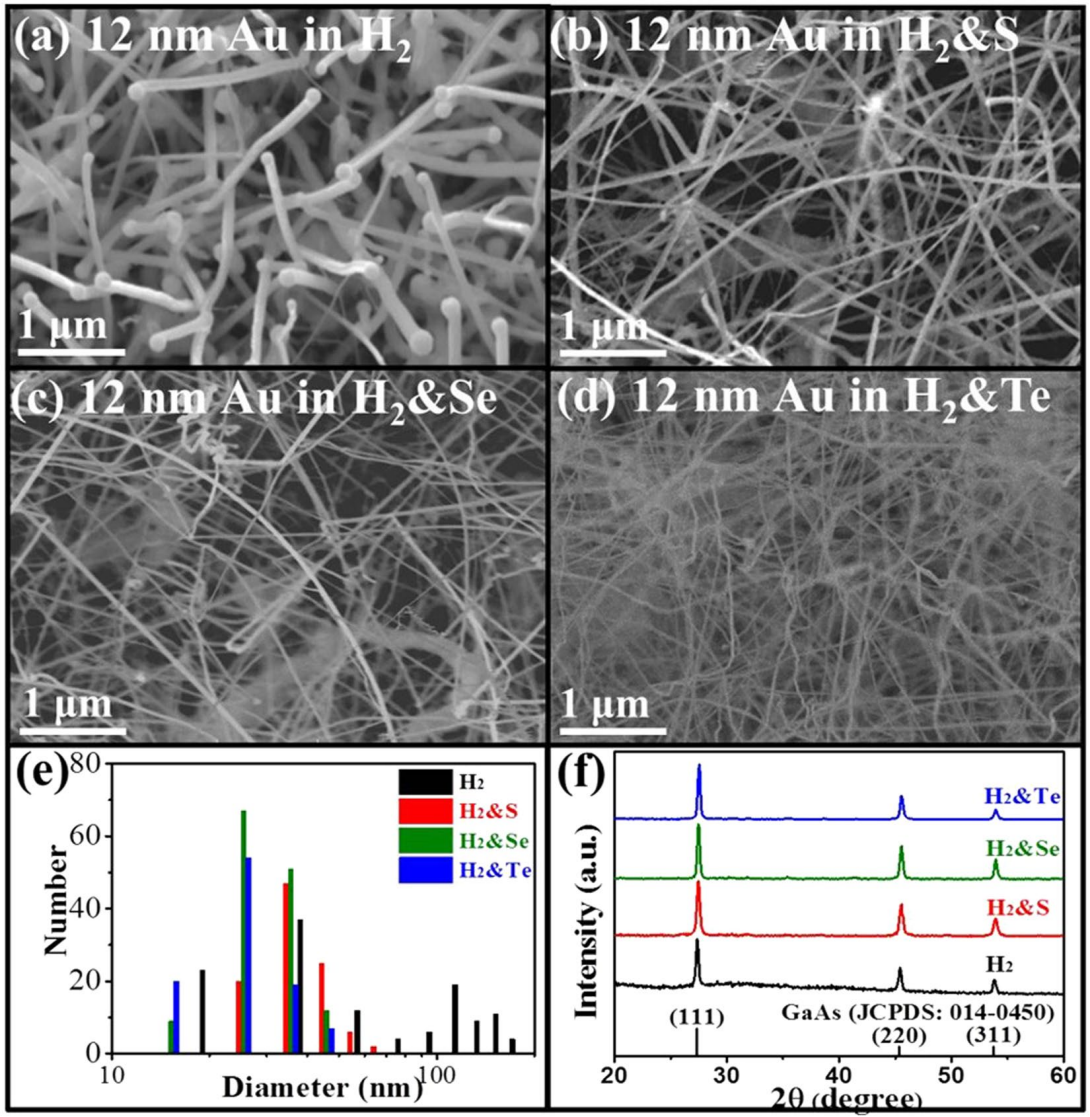
Morphology, diameter comparison and structural characterization of all GaAs NWs prepared by 12 nm thick Au catalyst films in H_2_ atmosphere. SEM images of GaAs NWs prepared (**a**) without any chalcogen passivation, (**b**) with S passivation, (**c**) with Se passivation and (**d**) with Te passivation during the NW growth; (**e**) diameter distribution of NWs grown without and with the chalcogen passivator; (**f**) XRD patterns of the as-prepared GaAs NWs.

In the past works, the mean diameter of GaAs NWs obtained can be tailored by controlling the thickness of Au catalyst films ranging from 0.1–12 nm^41,42^. However, when thick Au catalyst films, such as the nominal thickness of 6 and 12 nm are used, the grown NWs would have large diameter distributions spanning from tens to hundreds of nm. Taking the example of GaAs NWs grown by 12 nm thick Au catalyst films, both thin and thick NWs are grown on the same substrate as depicted in SEM image in Fig. 1a. In contrast, the NWs are grown much thinner and more importantly more uniform as illustrated in SEM images in Fig. 1b–d where chalcogens are adopted as the *in-situ* passivator during the NW growth. To quantify the diameter distribution, diameters of over 100 NWs are measured from their corresponding TEM images and the histogram is compiled in Fig. 1e. It is noticed that all S, Se and Te assisted growth can effectively reduce the diameter distribution. Specifically, Te can substantially decrease the average NW diameter from 76.1 ± 49.9 nm (without passivation) to 26.2 ± 6.6 nm (with passivation) as listed in Table 1. In the meanwhile, all the NWs grown with and without chalcogen passivation have the same cubic zincblende structure as shown in the XRD patterns in Fig. 1f (JCPDS Card No. 014-0450). Therefore, the *in-situ* chalcogen passivation is observed to effectively reduce the NW diameters and their distribution for the enhanced NW synthesis without affecting their crystallinity.

**Table 1:** Diameter comparison of GaAs NWs prepared by using different chalcogens as *in-situ* surface passivators (unit: nm).

Au catalyst	H_2_	H_2_ & S	H_2_ & Se	H_2_ & Te
6.0	64.1 ± 17.2	29.5 ± 8.5	28.4 ± 6.9	25.4 ± 6.3
12.0	76.1 ± 49.9	36.9 ± 8.6	29.9 ± 7.3	26.2 ± 6.6

**Figure 2:**
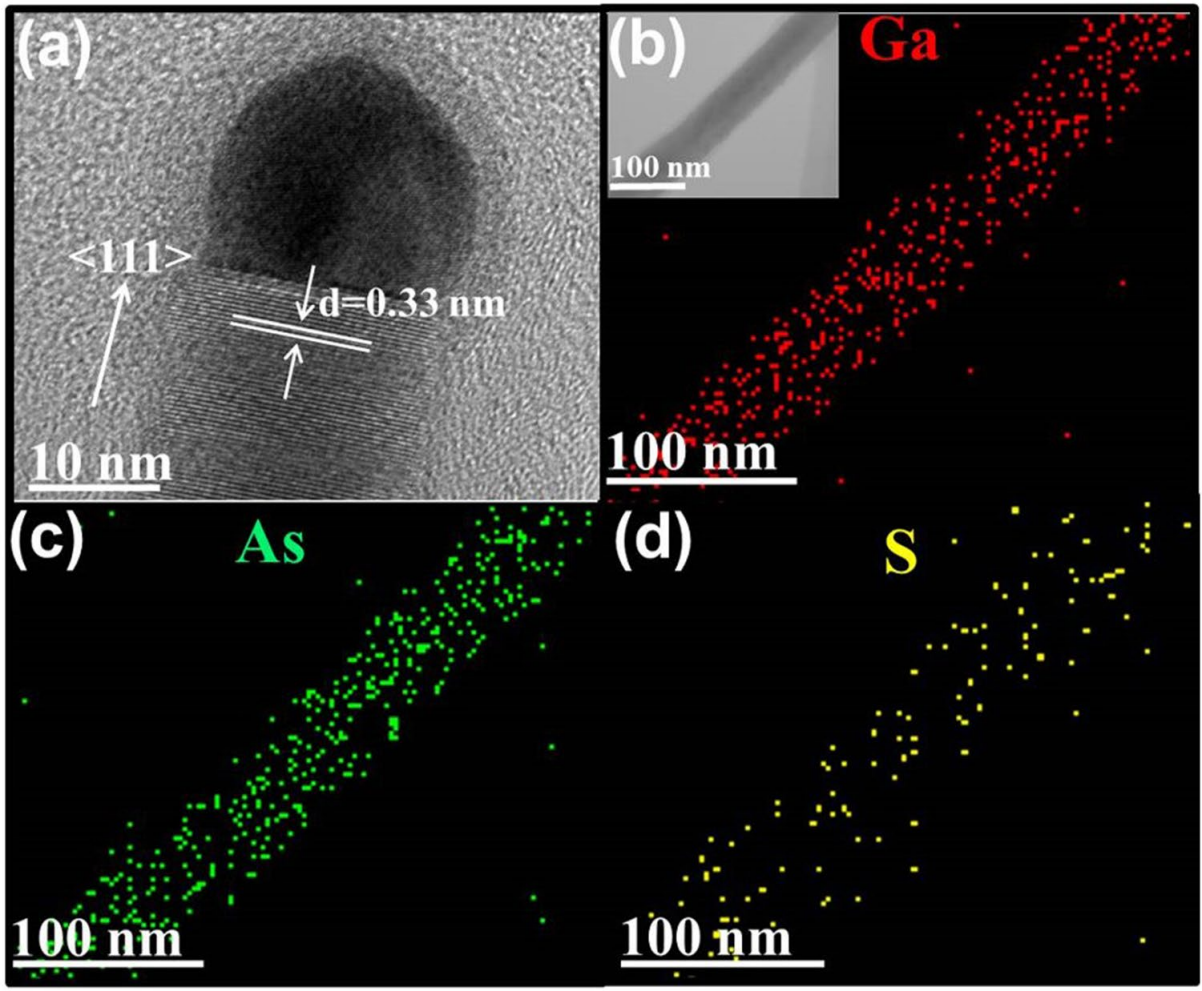
Electron microscopy characterization of thin sulfur-assisted grown GaAs NWs. (**a**) High-resolution transmission electron microscope (HRTEM) image of a representative NW, illustrating the catalyst seed/body region. The diameter is ~23 nm with the growth direction of <111>; (**b**–**d**) EDS elemental mappings of Ga, As and S, respectively. The inset is the TEM image of an individual NW for collecting the EDS spectra.

In order to further verify the crystallinity and the chemical stoichiometry of chalcogen-assisted GaAs NWs, high-resolution TEM (HRTEM) along with energy-dispersive X-ray spectroscopy (EDS) are performed taking the sulfur-grown GaAs as a representative example as illustrated in Fig. 2. One can witness that the typical NW surface is smooth with a diameter of 23 nm, and the zinc blende structure NW is single crystalline with a dominant growth axis of <111> direction. This preferential growth orientation is expected due to the lowest free surface energy in {111} planes of cubic III-V NW materials. As shown in Figure SI1 (Supplementary information, SI), the growth orientation of Se-GaAs NWs, with the diameter smaller than 100 nm, also follows <111>directions. Importantly, there is not any noticeable crystal defects, such as stacking faults and twin boundaries, existed in the NWs^43–45^. Furthermore, the observed hemispherical catalytic tip in Fig. 2a confirms the vapor-liquid-solid (VLS) growth mechanism in the adopted CVD technique. The stoichiometry of the catalyst tip and the NW body is then confirmed by the EDS, demonstrating that the NW body has a balanced stoichiometry of Ga:As ~ 1:1 with a catalyst tip of Au_2_Ga. All these findings are consistent to the highly crystalline GaAs NWs obtained previously without using chalcogen passivation^5,19,42^. Moreover, based on the elemental mapping in Fig. 2b–d, it should also be noted that all NW constituents (i.e. Ga and As atoms), including the S passivator, are uniformly distributed along the NW without any stoichiometric defects such as Ga or As clusters, indicating the well-controlled surface morphology, crystallinity and balanced stoichiometry of GaAs NWs obtained in this S assisted CVD technique.

**Figure 3:**
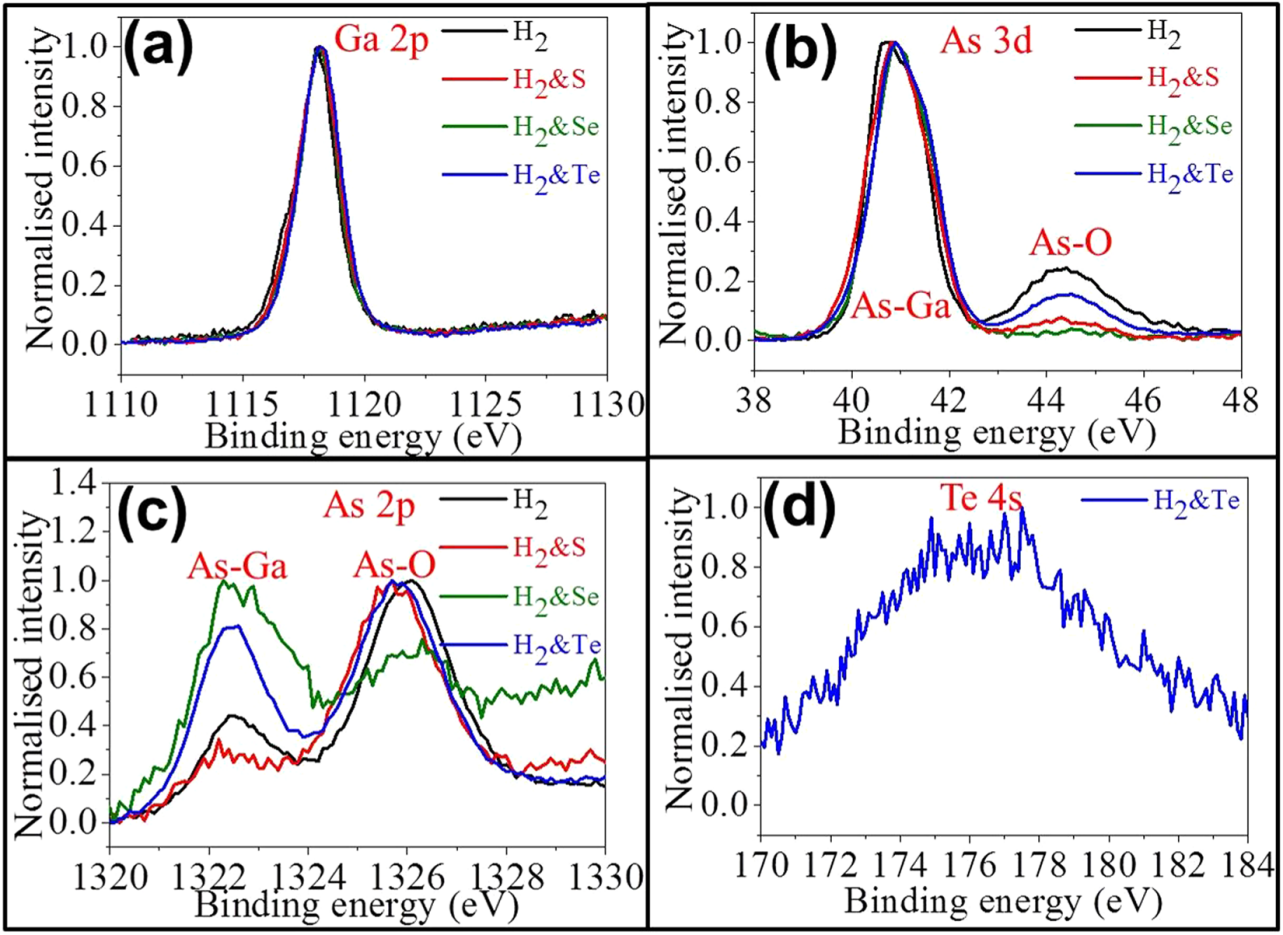
Surface elemental analysis of all as-prepared GaAs NWs. (**a**) Ga 2p bonding region; (**b**) As 3d bonding region; (**c**) As 2p bonding region and (**d**) Te 4 s bonding region.

To shed light onto the role of *in-situ* chalcogen passivation on the NW surface, XPS is performed on all as-prepared GaAs NWs with the results shown in Fig. 3. According to Figs 3a and SI2a, the single profile centered at 1118.1 eV can be decomposed into two peaks of 1118.2 eV and 1117.7 eV, relating to Ga-O and Ga-As bonds^46^, respectively. Obviously, the peak fitting of Ga 2p did not show significant changes as compared with those of As 3d and As 2p spectra in Figs 3b,c, SI2b,c. In the literature, the surface dangling bond is usually found to be related with As^47^, while the surface passivation of GaAs would induce more As related bonds instead of the ones of Ga. Since there is not any noticeable difference in the Ga 2p spectra of NWs attained with and without chalcogen passivators, it suggests that no chalcogen is combined with Ga to form stable covalent bonds on the NW surface. Nonetheless, as depicted in the corresponding As 3d spectra in Fig. 3b, there are two dominant peaks centered at 40.7 eV and 44.3 eV for the NWs grown without using chalcogen passivation, and these two peaks can be assigned to the construction of As-Ga and As-O bonds, respectively^48,49^. At the same time, it is interesting to find that the peak intensity of As-O bonds (i.e. centered at 44.3 eV) becomes weaker with the use of S and Te passivators, and totally diminished by using Se passivators during the NW growth. This change can be designated to the passivating function of S, Se and Te by contributing As-S (As_2_S_5_)^50^, As-Se (As_2_Se_3_)^51^ and As-Te (As_2_Te_3_)^51^ bonds in reducing the formation of native oxide on GaAs NWs and minimizing the uncontrolled radial NW growth as given in the previous literature report^44^ and Fig. SI2. In this case, Se is found to act as the best surface passivator among all chalcogens here, which can be probably attributed to the optimal size and electronegativity matching between Se and As constituents as compared to the ones of S and Te. Meanwhile, the Te passivator is also observed to yield a totally different behavior in addition to the weak passivating effect on GaAs NWs. As shown in Fig. 3c, XPS is performed in the As 2p region and these As 2p electrons have higher binding energy than the ones of As 3d; therefore, they would have the shorter emission length through the surface, which is more surface sensitive to the outmost 1-2 atomic layers. It is obvious that the outmost surface As atom is preferred to bond with oxygen as all As-O bonds (i.e. centered at 1326 eV) have the relatively high intensity on the NWs grown with S (i.e. red curve), Te (i.e. blue curve) and without any passivation (i.e. dark curve). The intensity of the As-O peak is considered comparatively weak for the Se passivated NWs (i.e. green curve) owing to the strongest surface passivation effect as discussed in Fig. 3b. However, the As-O peaks are also perceived to have a blue shift for the chalcogen passivated NWs, which is attributable to the establishment of As-S, As-Se and As-Te bonds as shown in the corresponding deconvoluted XPS peaks in Fig. SI2. Notably, since Te has the relatively lower electronegativity (2.1) than the ones of Se (2.55), S (2.58) and even As (2.18)^52^, Te is expected to perform as an electron donor when the Te-As bond is constructed on the GaAs NW surface in addition to simply passivate the reactive As constituents. This can be further ascertained by inspecting the Te 4 s spectrum in Fig. 3d, where the peak at 176.1 eV is attributed to Te^6+^ existing in its higher oxidizing state^53^. Also, as Te has the bulkiest size among all chalcogens investigated, the inefficient surface packing of Te passivators is anticipated on the NW surface, yielding the ineffective surface passivation and the significant As-O peak observed in Fig. 3b. As a result, during the *in-situ* surface passivation, Te would be functioned as a shallow donor attaching onto the surface of GaAs NWs while surface As would still be oxidized to give Ga-As, Te-As and As-O bonds, contributing to the formation of thinner and more uniform NWs along with the noticeable surface native oxide layer. On the other hand, it is worth to point out that the group VI powders were firstly evaporated at low temperautre of 200–300 °C before GaAs powder got vaproized (700–800 °C). Therefore, there is only the residual group VI element vapor in the growth system, which leads only to the surfactant effect instead of effectively doping into the nanowire lattice^54,55^. In the meanwhile, the doping can be more effective for VS lateral growth as compared with the VLS axial growth by Au catalysts, which might be attributed to the small solubility of the dopants in Au catalysts^47^. As a result, the group VI elements are more probable to behave merely as the surfactant but to only contribute the minor doping by Te.

**Figure 4:**
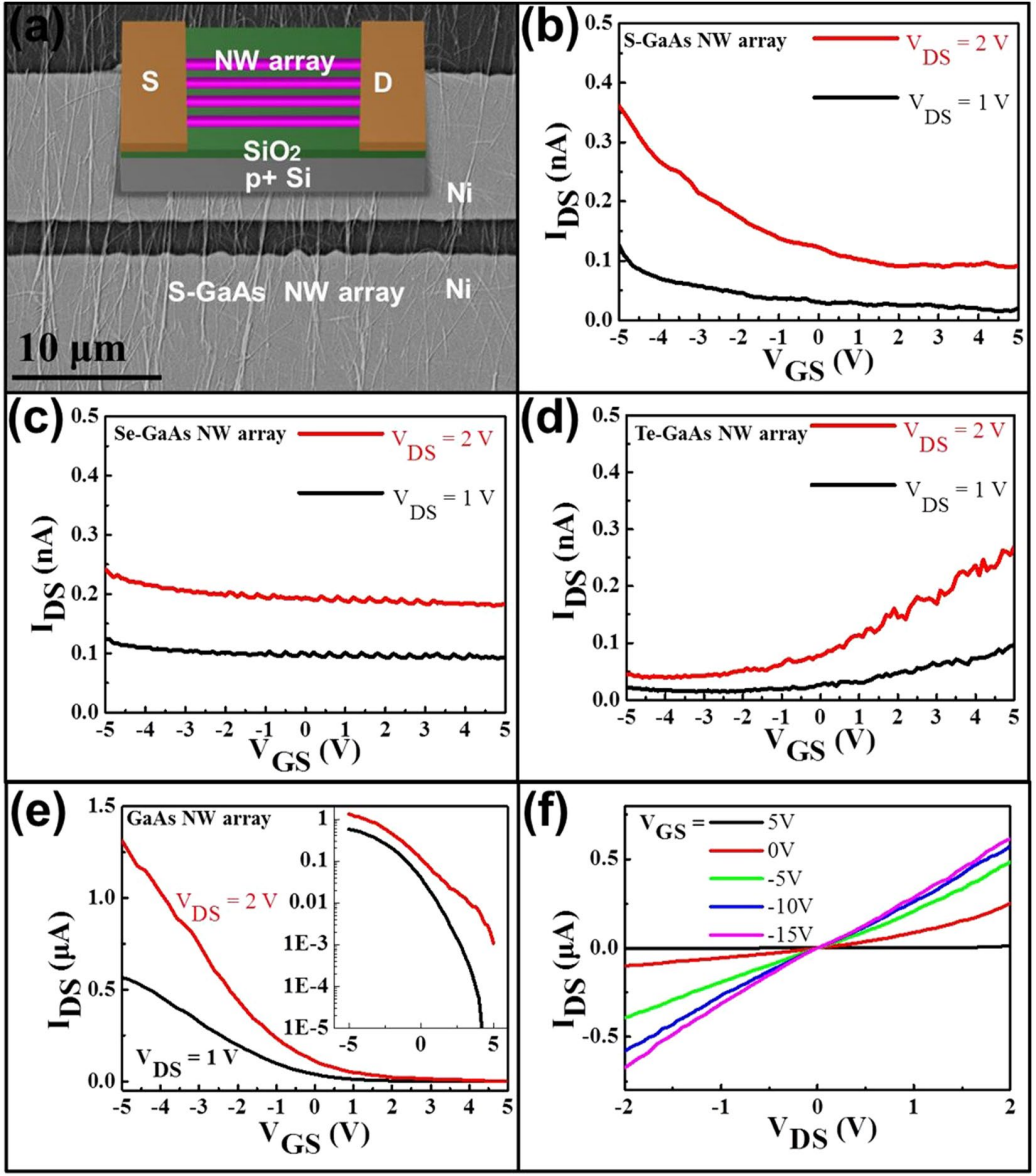
Electrical properties of the as-prepared GaAs NW arrays. (**a**) SEM image of a representative NW arrayed FET with the channel grown with the use of S as the passivator. Inset shows the illustrative device schematic of the parallel arrayed NWFET; (**b**–**d**) transfer characteristics of parallel arrayed FETs of GaAs NWs prepared by using S, Se and Te as passivators respectively; (**e**,**f**) are the I_DS_-V_GS_ and I_DS_-V_DS_ curves of GaAs NW arrayed FETs, accordingly. The NWs are grown without using any chalcogen passivation but with the similar NW diameter of 20–30 nm employing 2.5 nm thick Au catalyst films.

Apart from the effect on the NW surface, hundreds of parallel NW arrayed FETs are also fabricated in the well-known back-gated geometry with NW channels passivated with different chalcogen in order to evaluate the influence of various chalcogen passivation on electronic transport properties of GaAs NWs, as shown in Fig. 4. In this work, the NW to NW variation is minor in the as-fabricated NWs array, owing to the growth orientation of the as-prepared GaAs NWs is <111> with the diameters are smaller than 100 nm. On the other hand, the single as-prepared GaAs NW FETs show very small current (~pA) in Fig. SI3. As a result, the electronic transport property of GaAs NWs array is studied here, instead of single NW. The NW arrays (containing 500~1000 NWs considering the 2~4 NW/μm density and the 250 μm length in Fig. 4a) are all prepared by a well-established contact printing technique to minimize the wire-to-wire variations^56^. Figure 4b–d illustrate typical transfer characteristics of the parallel arrayed devices made of GaAs NW channels surface passivated with S, Se and Te, respectively. Figure 4b shows a representative weak p-type electrical conductivity of S-GaAs NW arrays, similar to the results of (NH_4_)_2_S passivated H_2_ grown GaAs NWs as shown in Fig. SI4. When Se is employed as the passivator, the p-type characteristic diminishes to yield the insulator-like behavior, which is consistent to the XPS results that Se provides the best surface passivation in reducing the depletion effect of the surface states existed between GaAs NW cores and their native oxide shells (Fig. 4c). On the other hand, while Te is passivated onto the NW channels, the array exhibits the n-type conductivity that perfectly agrees with the above-discussed electron donating property of Te (Fig. 4d). In this case, simply by using different chalcogens of S, Se and Te as passivators attaching onto the NW surface, the conducting behaviors of GaAs NWs can be controlled successfully. For the same residual impurity level, all the as-prepared GaAs NWs are grown in the same CVD system, which results that there is not any intentional impurity or dopant introduced during the NW growth. At the same time, all of the device fabrication is performed under the same condition, while the quasi-ohmic contact of Ni/GaAs is also shown in Fig. SI5. As a result, the electrical p-n switching of GaAs NWs is indeed contributed by choosing different chalcogens as the passivator.

To serve as a control, GaAs NWs with similar diameter of 20–30 nm by utilizing the thinner Au catalyst film of 2.5 nm are also grown without any *in-situ* chalcogen passivation. As shown in Fig. 4e,f, the NW arrayed FET is confirmed to display clearly the p-type conductivity with the larger ON current than the case of S-GaAs NW arrayed FETs. However, when they are measured under dark condition, the ON current drops to the nA level as shown in Fig. SI6. This proves again that if the GaAs surface is not passivated, the light induced carriers would occupy the surface states, leading to the different conductivity observed. The effect of incident light on the electronic transport properties of III-V NWs is complicated, such as the photo-gating effect and etc, in which all these will be studied in more detail in the near future. Anyhow, all these results are in the good consistence with previous results that the p-type conductivity of these thin GaAs NWs is contributed from the acceptor-like surface traps located at the interface between NW and its native oxide layer without any intentional doping^19^. The corresponding less p-type or insulating-like behavior (~3 orders of magnitude lowered current) of NWs is resulted by the diminishment of surface oxide (i.e. carrier depletion effect) due to the efficient Se passivation, whereas the n-type characteristic is brought about by the surface Te doping along with its passivating effect on GaAs NWs.

**Figure 5:**
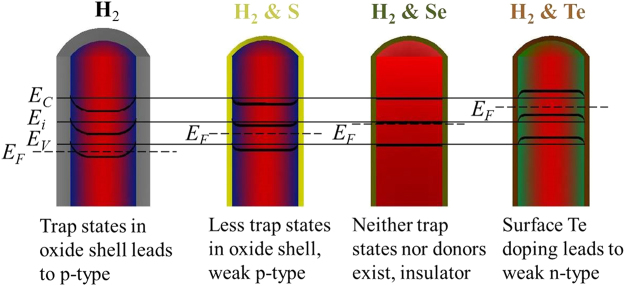
Cross-sectional view of thin GaAs NWs grown without or with different *in-situ* chalcogen passivation and the corresponding equilibrium energy band diagram at the zero gate bias.

Schematics along the radial direction of NWs with the equilibrium energy band diagram at the zero gate bias are shown in Fig. 5. As discussed in the past^19^, the native surface oxide shell layer would induce the acceptor-like interface trapping defects reducing electron concentrations in the NWs and generate the corresponding surface band bending. For the thin NWs with high crystal quality, less free electrons are generated by crystal defects and the depletion region extends over the entire volume of NWs (~40 nm diameter) because of their extremely high aspect ratio (i.e. large surface-to-volume ratio). This would shift the energy bands with respect to the Fermi level (E_F_), yielding inversion and p-type characteristics along the acceptor-like oxide/NW interface trapping states. As discussed in XPS results, when passivators of S and Se are employed in the NW growth, the NW surface atoms are then connected to the chalcogen, weakening the role of the native oxide shell, pushing the band to yield the weaker p-type conductivity and even insulator-like behavior, respectively. In contrast, when Te is adopted to passivate the NWs, it contributes to the surface doping effect by forming Te-As bonds. These surface donors would donate more electrons than those consumed by the trapping states in the unpassivated oxide layer, moving the band to give a conduction behavior switch from p-type to n-type. It should be also noted that although it is tuned, the Fermi level should be very close to the intrinsic level (E_i_) because of the relatively small current (i.e. high resistivity) as shown in Fig. 4. Consequently, all these results indicate the effective *in-situ* passivation effect of chalcogens on GaAs NW growth and their corresponding electrical properties, being promising for further tailoring the surface states of III-V semiconductors for high-performance electronics and optoelectronics.

## Discussion

Owing to the large surface-area-to-volume ratio of nanowires, manipulation of their surface states becomes technological important and being investigated for various applications. In this work, a methodology to enhance the growth and to modulate electronic transport properties of GaAs NWs by using *in-situ* chalcogen passivation is systematically presented. All of the chalcogens are found to connect to surface As constituents by forming stable covalent bonds onto the NWs and hence, the carrier depleting effect of NW native oxide shells on the corresponding electronic transport behaviours would become weaker, resulting a conductivity switch form p-type to n-type in the fabricated NW parallel arrayed FETs. Understanding all these would be essential to design and control electrical characteristics of GaAs NW devices via surface passivation for their practical deployment. In the future, this controllable passivation scheme can also be extended to other III-V NW material systems for the improved synthesis and the advanced technological applications.

## Methods

### GaAs NWs growth

All of the chalcogen passivated GaAs NWs studied here are synthesized by utilizing the surfactant-assisted solid-source CVD method as reported previously^38–40^. In brief, the solid powders of GaAs (99.999% purity) and chalcogen (i.e. sulfur, selenium and tellurium with the purity of 99.99%) are used as the source precursors. A two temperature zone horizontal tube furnace, with one zone for the solid source (upstream) and another zone for the growth substrate (downstream), is employed as the reaction chamber while Au films (i.e. 6 nm and 12 nm in the nominal thickness) are utilized as catalysts for the NW synthesis. Initially, catalyst films are pre-deposited onto Si/SiO_2_ substrates (50 nm thick thermally grown oxide), and the substrates are placed in the middle of the downstream zone. The solid sources, GaAs and chalcogen powders, are placed in two separate boron nitride crucibles next to each other, with distances of 15 cm and 9 cm away from the growth substrate, respectively, as shown in Fig. SI7. It is noted that the chalcogen powder is actually placed in the middle between two zones. During the CVD synthesis, the source and substrate are heated to the pre-set temperatures, correspondingly. Hydrogen with the purity of 99.9995% purity is utilized as the carrier gas to deliver the thermally evaporated constituents to the downstream. Prior to heating, the pressure of the tube is pumped down to 3 × 10^−3^ Torr and then purged with H_2_ for 0.5 h. After the growth, the source and substrate are stopped heating together and the substrate is cooled down to room temperature under hydrogen environment. The optimal growth conditions of GaAs NWs are given in Table SI1 and Fig. SI8 in the Supplementary information.

### Material characterization

Surface morphologies of the grown NWs are examined using a scanning electron microscope (SEM, FEI Company, Oregon, USA/Philips XL30, Philips Electronics, Amsterdam, Netherlands) and a transmission electron microscope (TEM, Philips CM-20). Crystal structures are determined by collecting XRD patterns on a Philips powder diffractometer using Cu Kα radiation (λ = 1.5406 Å) and imaging with a high resolution TEM (HRTEM, JEOL 2100 F, JEOL Co., Ltd., Tokyo, Japan). Elemental mappings are performed using an energy dispersive x-ray (EDS) detector attached to the JEOL 2100 F to measure the chemical composition of the obtained NWs. The chemical state of the as-prepared GaAs NWs is examined by X-ray photoelectron spectroscopy (ULVAC-PHI5802). For the TEM studies, the NWs are first suspended in ethanol solution by ultrasonication and then drop-casted onto the grid for the corresponding characterization.

### Nanowire array FET fabrication and electrical measurements

The obtained GaAs NWs are then contact-printed on the SiO_2_/Si (50 nm thermally grown oxide) substrates to assemble NW parallel arrays as reported in the past^57^. All of the substrates were spin-coated with LOR and AZ5206 photoresist, and next went through standard photolithography and development, followed by Ni electrode deposition (50 nm) and lift-off. Electrical performances of fabricated NW parallel array FETs are finally characterized with a standard electrical probe station (with the microscope light intensity of ~30 mW cm^−2^) and Agilent 4155 C semiconductor analyzer (Agilent Technologies, California, USA).

## Electronic supplementary material

Supplementary Information
