# Supplementary material for: Chalcogen passivation: an in-situ method to manipulate the morphology and electrical property of GaAs nanowires
Source: Sci Rep. 2018 May 2;8:6928. doi: 10.1038/s41598-018-25209-x (PMC5932019; doi:10.1038/s41598-018-25209-x)
Supplement: Supplementary file 1 — Supplementary Information [file 41598_2018_25209_MOESM1_ESM.pdf]

Supplementary information for

Chalcogen passivation: an *in-situ* method to manipulate morphology and electrical property of GaAs nanowires

Zai-xing Yang,[1,2#] Yanxue Yin,[2#] Jiamin Sun,[1,2] Luozhen Bian,[1,2] Ning Han,[3*] Ziyao Zhou,[4,5] Lei Shu,[4,5] Fengyun Wang,[6] Yunfa Chen,[3] Aimin Song,[1,7] Johnny C. Ho[4,5,8*]

[1]Shenzhen Research Institute of Shandong University, Shenzhen, 518057, P. R. China

[2]Center of Nanoelectronics and School of Microelectronics, Shandong University, Jinan, 250100, P. R. China

[3]State Key Laboratory of Multiphase Complex Systems, Institute of Process Engineering, Chinese Academy of Sciences, Beijing, 100190, P. R. China

[4]Department of Materials Science and Engineering, City University of Hong Kong, 83 Tat Chee Avenue, Kowloon, Hong Kong

[5]Shenzhen Research Institute, City University of Hong Kong, Shenzhen, 518057, P. R. China

[6]College of Physics and Cultivation Base for State Key Laboratory, Qingdao University, Qingdao, 266071, P. R China

[7]School of Electrical and Electronic Engineering, University of Manchester, Manchester M13 9PL, UK

[8]State Key Laboratory of Millimeter Waves, City University of Hong Kong, 83 Tat Chee Avenue, Kowloon, Hong Kong

[#] These authors contributed equally to this work.

E-mail: nhan@ipe.ac.cn and johnnyho@cityu.edu.hk

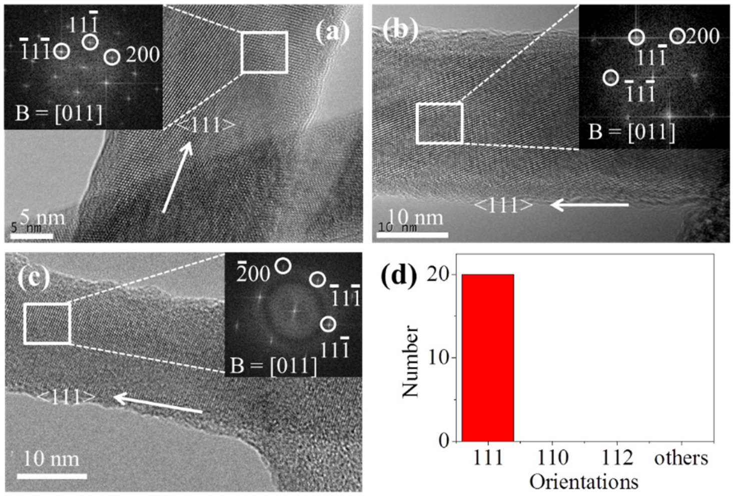

Figure SI1. HRTEM, FFT images and growth orientation statistics of Se-GaAs NWs.

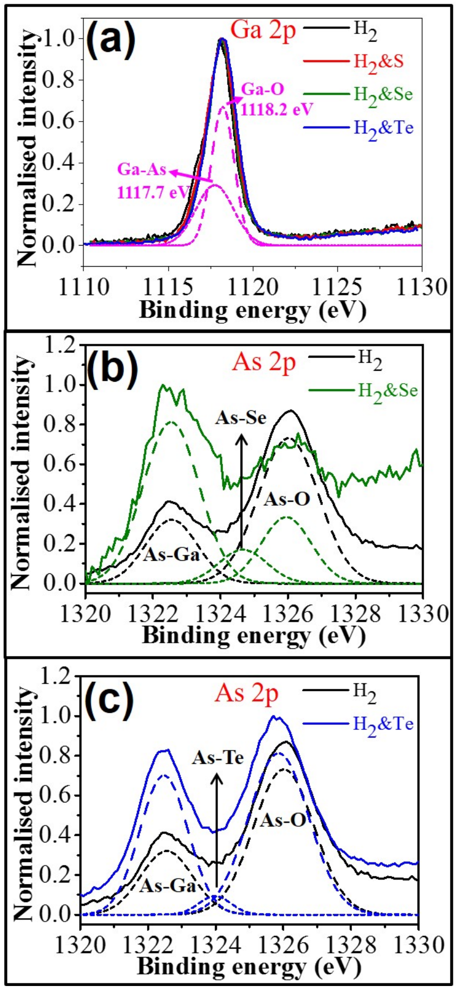

Figure SI2. Surface elemental analysis of the as-prepared Se- and Te-passivated

GaAs NWs. (a) Ga 2p bonding region of the as-prepared GaAs NWs. The pink dotted line shows the Gauss-Lorentz fitted spectra of the Ga 2p bonding region of the S-assisted GaAs NWs. (a, b) Gauss-Lorentz fitted spectra of the As 2p bonding region of the Se- and Te-assisted NWs. All of the correlation coefficient is $r^2>0.99$, respectively. In fact, for the Ga2p spectra in Figure 3a, there are Ga-O (1118.2 eV) and Ga-As (1117.7 eV) related peaks as shown in Figure SI2a. Obviously, they did not show significant changes as compared with those of As3d spectra in Figure 3b and c. In the literature, the surface dangling bond is usually found to be related with As[1], while the surface passivation of GaAs would induce more As related bonds instead of the ones of Ga. From Figure SI2b & c, it is obvious that As-Se bond (As_2Se_3, centered at 1324.7 eV)[2] and As-Te bond (As_2Te_3, centered at 1324.0 eV)[2] can be found in Gauss-Lorentz fitted spectra of the As 2p bonding region of the Se- and Te-assisted NWs, respectively.

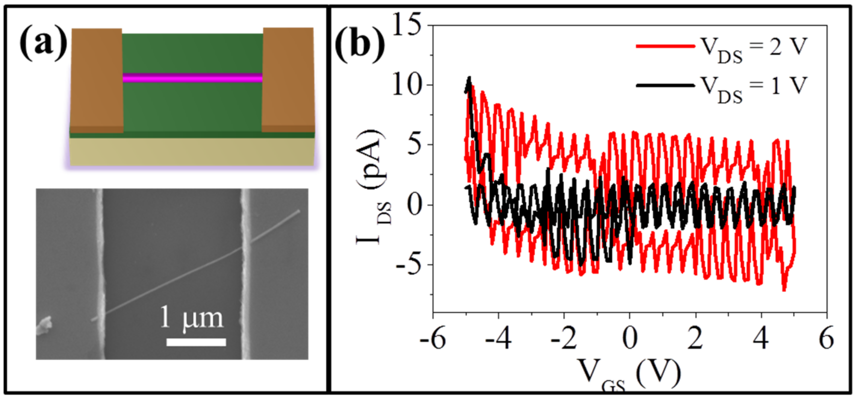

Figure SI3. The typical single Se-GaAs NW (a) FET device and (b) IV curves.

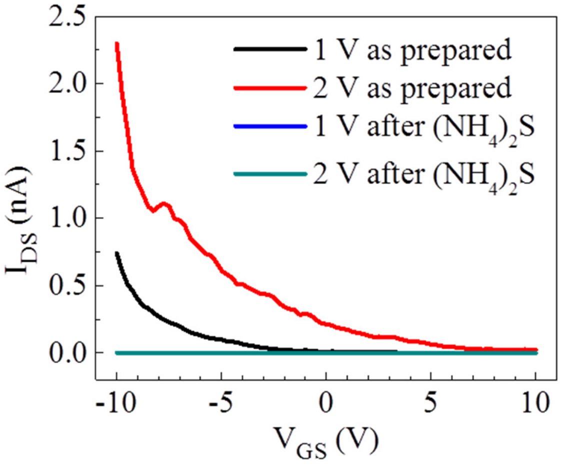

Figure SI4. The IV curves of GaAs NWs grown in H_2 atmosphere and then passivated in $(NH_4)_2S$ solution.

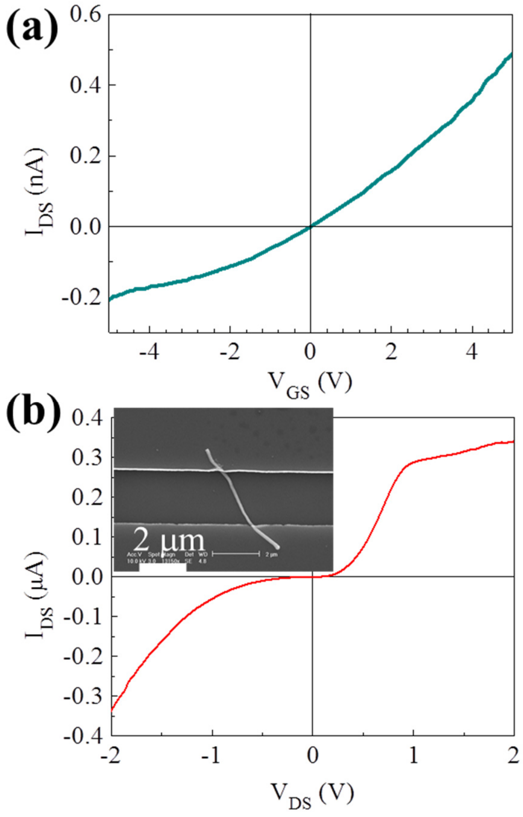

Figure SI5. The IV curves of GaAs NWs. (a) Se-GaAs and (b) thick GaAs NWs grown in H_2 in the previous study[3,4].

In our previous study, the thick and defective GaAs NWs are n-type to metallic, which showed Schottky contact with GaAs as shown in Figure SI5 (the asymmetric behavior is attributed to the AuGa catalyst tip beneath one electrode[5]). However, the Schottky barrier is not too high to be ~0.3-0.5 eV. Therefore, the Ni-GaAs contact has not too high Schottky barrier if any, and it would be measurable in the -5 V ~ 5V range measurement in Figure SI5a.

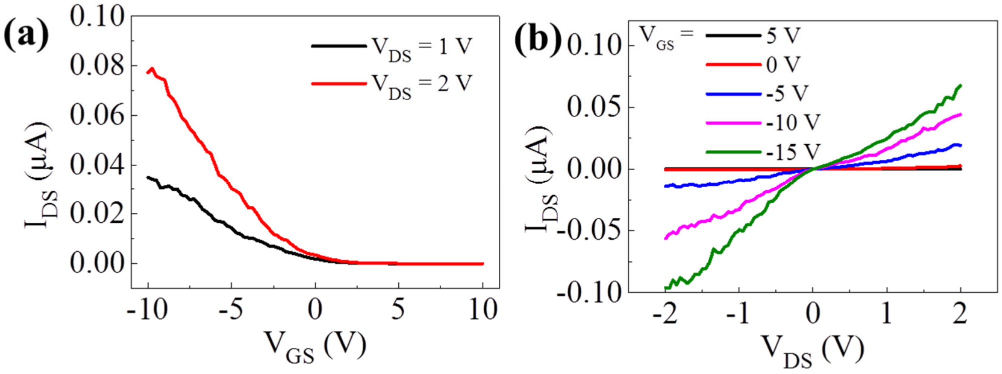

Figure SI6. The typical GaAs NW array FET measured under dark condition (NWs grown without in-situ chalcogen passivation). (a) $I_{DS} – V_{GS}$ and (b) $I_{DS} – V_{DS}$ curves.

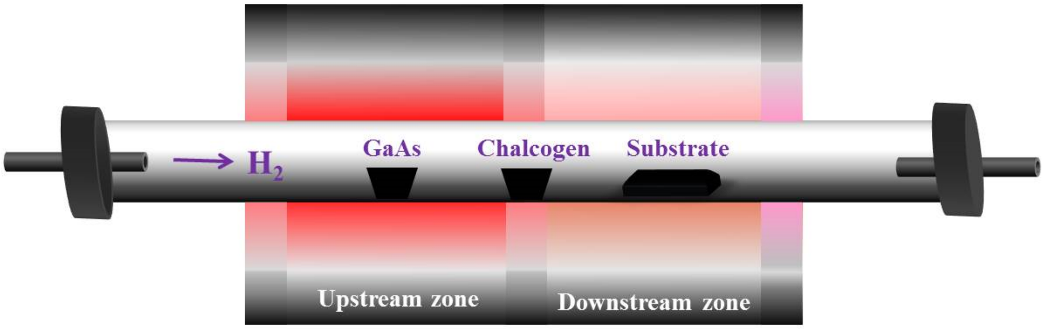

Figure SI7. Experimental setup of the chalcogen assisted GaAs NW growth.

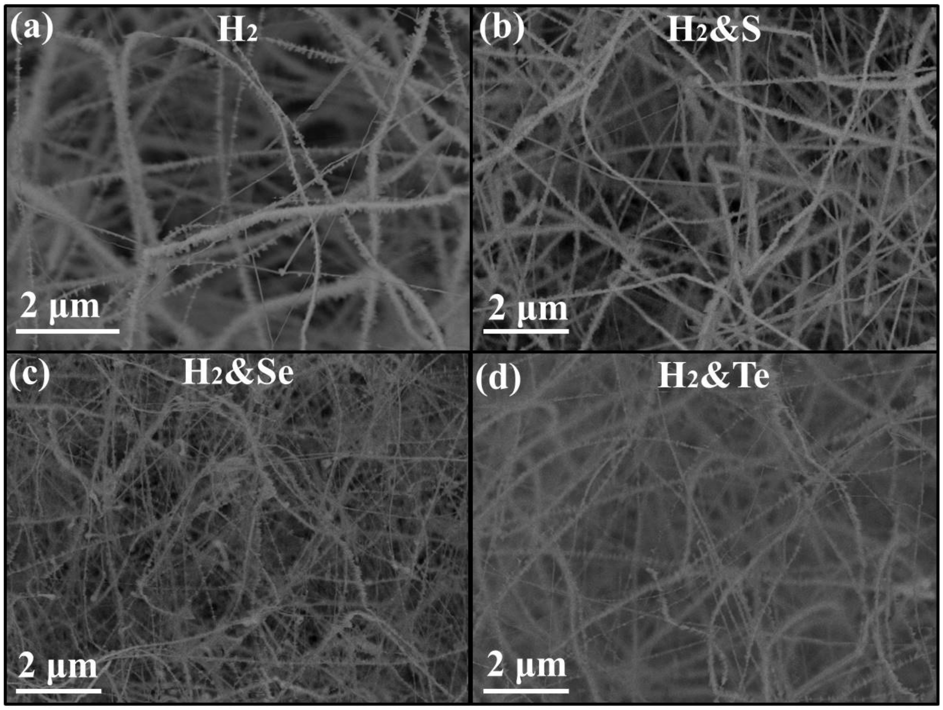

Figure SI8. The morphology of GaAs NWs prepared beyond the optimal growth conditions. SEM images of GaAs NWs prepared (a) without chalcogen, (b) with sulfur, (c) with Se, and (d) with Te in the NW growth process.

Beyond the optimal growth conditions listed in Table S1, heavy coatings are found on the surface of as-prepared GaAs NWs.

Table S1. The optimal growth conditions of as-prepared GaAs NWs performed in this study

Samples	Source Materials	Au Catalyst films thickness (nm)	Source Temperature (^{o}C)	Substrate Temperature (^{o}C)	Growth Time (min)	H_2 Flow (sccm)
S1	1g GaAs	12	900	600	60	200
S2	1g GaAs	6	900	600	60	200
S3	1g GaAs	0.5	900	600	60	100
S4	0.6g GaAs and 0.6g S	12	820	600	60	100
S5	0.6g GaAs and 0.6g S	6	820	600	60	100
S6	0.5g GaAs and 0.5g Se	12	850	600	60	200
S7	0.5g GaAs and 0.5g Se	6	850	600	60	200
S8	0.4g GaAs and 0.5g Te	12	850	580	60	300
S9	0.4g GaAs and 0.5Te	6	850	580	60	300

REFERENCES

1 Perea, D. E. *et al.* Direct measurement of dopant distribution in an individual vapour-liquid-solid nanowire. *Nat. Nanotechnol.* **4**, 315-319 (2009).

2 Bahl, M. K., Woodall, R. O., Watson, R. L. & Irgolic, K. J. Relaxation during photoemission and LMM Auger decay in arsenic and some of its compounds. *J. Chem. Phys.* **64**, 1210-1218, (1976).

3 Han, N. *et al.* Facile synthesis and growth mechanism of Ni-catalyzed GaAs nanowires on non-crystalline substrates. *Nanotechnology* **22**, 285607 (2011).

4 Han, N. *et al.* Controllable p-n switching behaviors of GaAs nanowires via an interface effect. *ACS Nano* **6**, 4428-4433 (2012).

5 Han, N. *et al.* GaAs nanowire Schottky barrier photovoltaics utilizing Au-Ga alloy catalytic tips. *Appl. Phys. Lett.* **101**, 013105 (2012).
